# Non-Volatile Metabolic Reprogramming and Sensory Evolution of Anhua Qianliang Tea During Long-Term Storage

**DOI:** 10.3390/foods15132376

**Published:** 2026-07-03

**Authors:** Mengzhen Xia, Zhichao Lin, Meihui Huang, Ju Zhou, Guohe Chen, Jianan Huang, Zhonghua Liu, Chao Wang

**Affiliations:** 1 Key Laboratory of Tea Science of Ministry of Education, Hunan Agricultural University, Changsha 410128, China; 18779354224@163.com (M.X.); linzhichao0101@163.com (Z.L.); 18674741735@163.com (M.H.); 15772797307@163.com (J.Z.); chenguohe1110@126.com (G.C.); jian7513@hunau.edu.cn (J.H.); larkin-liu@hotmail.com (Z.L.); 2 National Research Center of Engineering and Technology for Utilization of Botanical Functional Ingredients, Changsha 410128, China; 3 Tea Cultivar Innovation Center, Yuelushan Laboratory, Changsha 410128, China; 4 State Key Laboratory of Tea Plant Germplasm Innovation and Resource Utilization, Hunan Agricultural University, Changsha 410128, China; 5 Key Laboratory for Evaluation and Utilization of Gene Resources of Horticultural Crops, Ministry of Agriculture and Rural Affairs of China, Hunan Agricultural University, Changsha 410128, China

**Keywords:** Anhua Qianliang tea, long-term storage, physicochemical analysis, untargeted metabolomics, sensory evaluation

## Abstract

Anhua Qianliang tea, a traditionally crafted dark tea, is highly prized for its unique sensory properties that develop and improve during long-term aging. To elucidate the material basis driving the quality transformation of Anhua Qianliang tea during prolonged storage, samples aged for 2 to 25 years were investigated using physicochemical analysis, untargeted metabolomics, and sensory evaluation. Prolonged storage reduced total polyphenols, free amino acids, and most catechins, whereas alkaloids remained relatively stable. Phenylpropanoids and polyketides, organic acids and derivatives, and lipids and lipid-like molecules were the main metabolite classes contributing to storage-stage discrimination, accompanied by extensive remodeling of flavonoid, phenylpropanoid, amino acid-related, and central carbon metabolism. Sensory evolution was closely associated with coordinated changes in catechins, polyphenols, phenolic acids, amino acid-related compounds, and storage-responsive metabolites. These insights significantly deepen the understanding of dark tea maturation and offer new perspectives for scientific evaluation and industrial standardization.

## 1. Introduction

Anhua Qianliang tea (QLT) is one of the most representative steamed roll tea products within Anhua dark tea, a typical compressed dark tea category. Its unique processing steps including steaming, canning, bundling, and natural drying result in a cylindrical, tightly pressed structure, allowing the tea to continue undergoing quality transformation during subsequent storage [1,2]. Compared with other tea types, the quality development of dark tea and particularly Anhua dark tea depends heavily on extensive processing and prolonged aging. This aging process involves intricate physical and chemical transformations alongside microbial activity and thereby substantially alters the tea leaves’ color, taste profile, and overall sensory attributes [1,3]. In recent years, tea storage has been recognized as a dynamic process that remains incompletely understood and imprecisely regulated. The transformation of endogenous components is driven by multiple factors including duration, temperature and humidity, oxygen availability, and light exposure [2]. Therefore, elucidating the material basis underlying the quality evolution of QLT during long-term storage holds considerable significance for scientific quality assessment, vintage authentication, and high value utilization.

From a compositional standpoint, Anhua dark tea is abundant in polyphenols, flavonoids, catechins, alkaloids, amino acids, and phenolic acids as well as other non-volatile constituents. Collectively these substances constitute the primary chemical foundation for the taste attributes of the tea infusion including bitterness, astringency, body, and aftertaste [2,4]. Multi omics analyses have demonstrated that Anhua dark tea differs markedly from other traditional teas in its profile of non-volatile constituents including flavonols, amino acids, phenolic acids, and aldehydes. The unique compact structure of Anhua dark tea and its subsequent storage further increase the complexity of compositional transformations [1]. Previous investigations have established that dark tea undergoes a series of reactions during processing and fermentation including catechin degradation, oxidation, condensation, structural modification, and glycosylation. These transformations are intimately linked to the development of its distinctive flavor profile [5]. Therefore, a systematic elucidation of the chemical transformation patterns occurring during QLT storage based on non-volatile metabolic analysis is essential for understanding the mechanism of quality development during aging.

In recent years research on the storage quality evolution of dark tea and aged tea has expanded considerably. Metabolomics investigations have demonstrated that prolonged storage induces marked alterations in primary polyphenols, catechins, and other non-volatile metabolites in tea and consequently drives the sustained remodeling of taste quality [6,7]. For example, investigations of Keemun black tea storage have indicated that the predominant polyphenols undergo degradation during aging whereas caffeine and theobromine remain relatively stable [8]. Further investigations on Tibetan tea have demonstrated that samples corresponding to different storage years exhibit distinct clustering patterns and that key differential metabolites capable of discriminating among storage periods can be identified [9]. Simultaneously, the integration of sensory quantitative descriptive analysis (QDA) and metabolomics is emerging as a vital strategy for elucidating tea flavor formation [10]. Recent studies have integrated QDA with large scale quantitative metabolomics, nontargeted metabolomics, and molecular sensory analysis to elucidate the associations between specific taste attributes and non-volatile metabolites [11,12]. Although metabolomics and conventional chemometric approaches effectively reveal overall differences among tea samples subjected to varying storage durations, accurately identifying linear evolutionary relationships and key discriminant variables within high dimensional datasets remains challenging [1,6].

However, systematic studies on the long-term evolution of non-volatile quality in QLT remain limited. In this study, QLT samples with different storage years were investigated through physicochemical analysis, catechin quantification, UPLC-Q Exactive-Orbitrap-MS-based untargeted metabolomics, and quantitative descriptive sensory evaluation. These approaches were further combined with multivariate statistics and metabolite-taste correlation analysis to elucidate storage-associated changes in non-volatile constituents and sensory attributes. The objectives were to characterize the stage-dependent evolution of non-volatile quality in QLT and clarify the coordinated changes among physicochemical traits, catechin fractions, differential metabolites, and sensory attributes.

## 2. Materials and Methods

### 2.1. Tea Samples

Nine Anhua Qianliang tea (QLT) samples corresponding to different production years (2000, 2005, 2007, 2011, 2013, 2016, 2019, 2021, and 2023) were obtained from Hunan Liyuanlong Tea Industry Co., Ltd., located in Anhua, Hunan, China. At the time of sampling, the corresponding storage durations were 25, 20, 18, 14, 12, 9, 6, 4, and 2 years, respectively. To simplify sample identification, each sample was coded according to its storage duration using the prefix Q. For example, the sample stored for 25 years was designated as Q25. All samples were produced by the same manufacturer following standardized industrial procedures and using consistent raw materials. The production process primarily included screening, steaming, basket filling, pressing, shaping, and natural drying. After production, the samples were stored under natural warehouse conditions in Anhua, Hunan, China. Specifically, this traditional storage practice was conducted in dry and well-ventilated brick warehouses shielded from direct sunlight. Within these facilities, the tea underwent aging under standard ambient oxygen and natural subtropical seasonal variations, with ambient temperatures ranging from approximately 5 to 32 °C and relative humidity ranging between 70% and 85%. Before analysis, all samples were kept in a dry, well-ventilated environment away from light and foreign odors. Each sample was then ground into powder, sieved, sealed in an aluminum foil bag, and used for subsequent physicochemical testing, targeted catechin quantification, and untargeted metabolomics. Due to the scarcity of long-term aged tea samples, all measurements were performed in triplicate to ensure analytical reproducibility.

### 2.2. Chemicals and Reagents

Gallic acid (GA), theobromine, theophylline, caffeine, (-)-epi-gallocatechin gallate (EGCG), (-)-epicatechin gallate (ECG), (-)-epi-gallocatechin (EGC), (-)-epicatechin (EC), (+)-catechin (DL-C), (+)-gallocatechin-3-O-gallate (GCG), standards was obtained from Sigma-Aldrich Co., Ltd. (St. Louis, MO, USA). Disodium phosphate dodecahydrate, potassium dihydrogen phosphate, methanol, Tin (II) chloride, glutamate and Sodium Carbonate were purchased from Shanghai Hushi Chemical Reagent & Analysis Instrument Co. Ltd. (Shanghai, China). Folin phenol, ninhydrin and acetic acid were provided by China National Pharma-ceutical Corporation (Shanghai, China). Mass spectrometry-grade acetonitrile and formic acid, used for UPLC-Q Exactive-Orbitrap-MS analysis, were purchased from Merck KGaA (Darmstadt, Germany).

### 2.3. Determination of Physicochemical Characteristics

The conventional chemical composition of QLT samples was determined according to the Chinese national standard methods. Total flavonoids, free amino acids (FAAs), and tea polyphenols (TPs) were measured following the national standard GB/T 8314-2013 [13]. The contents of catechins, gallic acid (GA), and alkaloids were quantified using high-performance liquid chromatography (HPLC, Shimadzu, Japan) following previously established methods [14]. All measurements were conducted in triplicate to ensure reproducibility.

### 2.4. QDA Sensory Evaluation

Sensory evaluation was performed by 12 well-trained panelists from Hunan Agricultural University (six males and six females, aged 23 to 61 years) according to the Chinese national standard “Methodology of Sensory Evaluation of Tea” (GB/T 23776-2018 [15]). Each assessor had completed more than 90 h of specialized training in QDA. Specifically, this training comprised basic taste threshold screening, semantic alignment of the six targeted attributes, and repeated intensity-scaling calibration using standard reference infusions to ensure panel consistency. For each sample, 3.0 g of tea leaves was infused with 150 mL of boiling water in a standard cupping set and steeped for 5 min to establish the overall sensory profile. QDA was then used to assess taste attributes. Based on the initial group consensus during training and with reference to previous studies and national sensory evaluation standards, six descriptive terms were ultimately determined, namely umami, sweetness, mellowness, bitterness, astringency, and kokumi [12,16]. Evaluations were performed independently in an odor-free sensory room maintained at 21 ± 1 °C. Approximately 5 mL of infusion was rated on a structured 5-point scale (0 indicating none, 3 moderate, and 5 very strong), with each sample assessed in triplicate on separate days. Final scores were calculated as the mean of all panelists’ ratings. Ethical approval for sensory evaluation was not required by the institute. The study followed relevant operational standards in China, which ensured voluntary participation, informed consent, and protection of participants’ rights and privacy.

### 2.5. Untargeted Non-Volatile Compound Analysis

The extraction protocol and UPLC-Q Exactive-Orbitrap-MS parameters followed a previously validated method for tea metabolomics [14,16]. Briefly, 0.5000 g of tea sample was weighed into 25 mL of 70% (*v*/*v*) methanol in water and ultrasonicated for 30 min, with shaking every 10 min. The mixture was then centrifuged at 12,000 g for 10 min at 10 °C. The supernatant was collected and filtered for subsequent analysis. Pooled quality control samples were used to assess the stability and reproducibility of the metabolomic analysis.

Metabolomic profiling was performed using an ultrahigh performance liquid chromatography system coupled to a Q Exactive Orbitrap mass spectrometer (Thermo Fisher Scientific, Waltham, MA, USA). Separation was performed on a Hypersil Gold C18 column (1.9 μm, 2.1 × 150 mm). The mobile phase consisted of 0.1% formic acid in water (solvent A) and 0.1% formic acid in acetonitrile (solvent B). The gradient program was as follows: 0–1.5 min, 0% B; 1.6 min, 5% B; 18.0 min, 18% B; 34.0 min, 95% B; 38.5 min, 0% B; and 42 min for re-equilibration. The flow rate was 0.3 mL/min, the injection volume was 2 μL, and the column temperature was 35 °C. The spray voltage was +3500 V in positive mode and −2500 V in negative mode. The sheath gas was set to 35 arb, the auxiliary gas to 10 arb, the ion transfer tube temperature to 325 °C, and the vaporizer temperature to 300 °C. The full scan MS1 resolution was 120,000 at *m*/*z* 100 to 1500. The data-dependent MS/MS resolution was 15,000. Normalized collision energies were set to 10%, 30%, 50%, and 70%. The scanning period was 3 s. Raw data from UPLC Q Exactive Orbitrap MS were processed using Compound Discoverer 3.3 software (Thermo Fisher Scientific, Waltham, MA, USA) for peak extraction and matching, yielding retention times and peak intensities. In this study, all metabolites were putatively annotated without the use of authentic chemical standards. Structural annotation was exclusively performed by matching accurate precursor masses and tandem mass spectrometry (MS/MS) fragmentation data against the PubChem database and the relevant peer-reviewed literature.

### 2.6. Statistical Analysis

All experiments were performed in triplicate, and the results are expressed as mean values ± standard deviation. The QDA radar chart and bar chart were generated using Origin 2024b software (OriginLab Corporation, Northampton, MA, USA). Variable importance in projection (VIP) values were obtained from orthogonal partial least squares-discriminant analysis (OPLS-DA) and partial least squares-discriminant analysis (PLS-DA), while the corresponding score plots and permutation tests were generated using the R package MetaboAnalystR 4.0. Heatmap analysis and principal component analysis (PCA) were also performed using MetaboAnalystR. Advanced Cor link was performed using the OmicStudio tools at https://www.omicstudio.cn/tool, accessed on 1 April 2026. Identified metabolites were annotated using the KEGG pathway database (https://www.kegg.jp/, accessed on accessed on 1 April 2026) integrated within MetaboAnalyst 6.0 (https://www.metaboanalyst.ca/home.xhtml, accessed on accessed on 1 April 2026), and the corresponding pathway visualizations were also generated on this platform. Differential metabolites were screened using the following criteria: VIP score ≥ 1, *p* < 0.05, and |log_2_FC| >1.

## 3. Results

### 3.1. Changes in Physicochemical Traits and Catechin Composition Across Storage Years

To systematically assess the fundamental alterations in non-volatile quality of QLT during prolonged storage a joint analysis of physicochemical indices and principal catechin related constituents was performed. As presented in Table S1, samples corresponding to different storage durations exhibited varying degrees of variation in total flavonoid content total polyphenol content free amino acid levels and major catechin fractions. These observations indicate that long-term storage substantially restructures the fundamental chemical composition of QLT. Previous investigations have indicated that tea storage constitutes not a simple static process but rather a complex progression driven by concurrent biological chemical and physical reactions [1,2].

**Figure 1:**
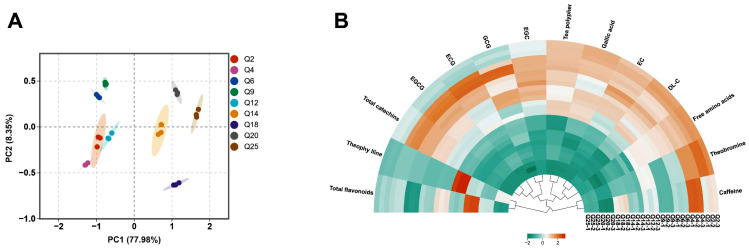
PCA and circular clustering analysis of physicochemical traits and catechin-related compounds in QLT with different storage years. (**A**) PCA score plot. (**B**) Circular hierarchical clustering heatmap. Orange and green indicate relatively high and low levels, respectively.

The PCA results further revealed the overall pattern of storage-related differences (Figure 1A). PC1 and PC2 explained 77.98% and 8.35% of the total variance, respectively. Samples from different storage years showed clear separation in the score plot, indicating that physicochemical parameters and catechin composition can effectively characterize the differences in the basic quality of QLT across storage stages. Overall, younger samples were mainly distributed in the negative region of PC1, whereas samples stored for longer periods were predominantly distributed in the positive region of PC1. Samples with intermediate storage durations occupied a transitional position, indicating that QLT undergoes distinct stage-dependent changes during long-term storage rather than simple linear decay. Similar storage-dependent stratification has also been reported in other studies on aged tea, in which samples often form an overall chemical gradient that gradually shifts from younger to older samples with increasing storage duration [8].

The circular hierarchical clustering heatmap further supports the above conclusion from the perspective of coordinated changes among variables (Figure 1B). Samples from different storage years showed clear grouping patterns in the heatmap, with higher similarity observed between adjacent storage years. In the heatmap, tea polyphenols, free amino acids, EGCG, ECG, EGC, GCG, and total catechins generally showed higher levels in younger samples but tended to decline in samples stored for longer periods. In contrast, caffeine, theobromine, and theophylline exhibited relatively minor changes, indicating storage-response patterns distinct from those of the major catechin components. Previous studies on the storage of Keemun black tea and green tea beverages have also shown that major polyphenols and catechins are often the most sensitive indicators of changes during aging, whereas alkaloids such as caffeine are relatively stable [8].

QLT undergoes significant remodeling of its basic non-volatile quality during long-term storage. Total polyphenols and free amino acids generally decreased with prolonged storage, indicating the continuous depletion of soluble quality-related substrates. Total flavonoids showed fluctuating changes, suggesting that flavonoid-related components may simultaneously undergo degradation, structural modification, and localized accumulation during storage [2]. Catechin components exhibited more typical stage-dependent changes, with EGCG, ECG, and EGC generally occurring at higher levels in younger samples and then gradually declining with prolonged storage. This pattern suggests that a brief phase of component rearrangement or enhanced extractability may occur during the early stage of storage, followed by sustained consumption and extensive transformation [7]. In contrast, EC more closely followed a continuous declining pattern, whereas gallic acid did not exhibit the stable accumulation trend commonly observed in certain aged tea systems, but instead showed a trajectory distinct from that of the major catechin backbone. Meanwhile, the magnitude of caffeine fluctuation was markedly smaller than that of catechins, indicating that its response to storage duration was relatively weak. Overall, these results suggest that the non-volatile quality evolution of QLT during long-term storage is characterized by systematic remodeling rather than by the synchronous increase and decrease of any single component.

Overall, the decline in total polyphenols, free amino acids, and major catechin components indicates that long-term storage is accompanied by the continuous depletion of basic quality-related substrates. In contrast, the relatively high levels of some catechins in younger samples suggest that a certain degree of component rearrangement and release may occur during the early stage of storage. Together, these results provide an important chemical basis for subsequent metabolomic analysis and investigations into flavor attribute evolution.

### 3.2. Global Metabolomic Discrimination Among Storage Years

**Figure 2:**
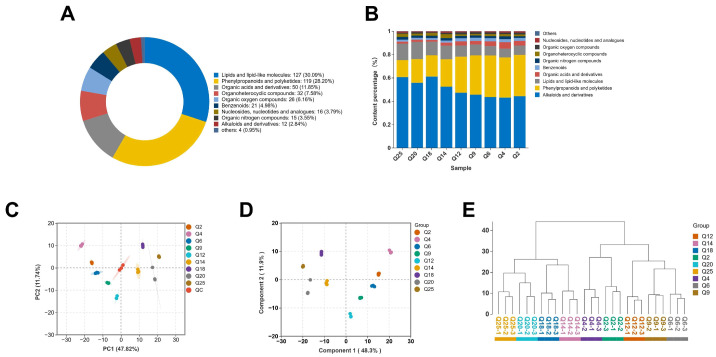
Classification and multivariate analysis of non-volatile metabolites in QLT with different storage years. (**A**) Pie chart of metabolite classes. (**B**) Relative stacked bar plot of metabolite classes in different samples. (**C**) PCA score plot. (**D**) PLS-DA score plot. (**E**) Hierarchical clustering dendrogram.

To systematically characterize changes in the non-volatile metabolic profile of QLT during long-term storage, untargeted metabolomic analysis was performed on samples from different storage years using UPLC-QExactive-Orbitrap-MS (Thermo Fisher Scientific, Waltham, MA, USA). A total of 422 non-volatile metabolites were detected and annotated, mainly including lipids and lipid-like molecules (127, 30.09%), phenylpropanoids and polyketides (119, 28.20%), organic acids and derivatives (50, 11.85%), organoheterocyclic compounds (32, 7.58%), organic oxygen compounds (26, 6.16%), benzenoids (21, 4.98%), nucleosides, nucleotides, and analogues (16, 3.79%), organic nitrogen compounds (15, 3.55%), alkaloids and derivatives (12, 2.84%), and a small proportion of other compounds (4, 0.95%) (Figure 2A). This result indicates that the non-volatile metabolic profile of QLT during long-term storage is highly complex, with lipids, phenylpropanoids, polyketides, and organic acid metabolites constituting the major chemical basis, in agreement with previous studies [1,4].

The composition of metabolite categories in samples stored for different years showed significant dynamic changes in relative abundance during storage (Figure 2B). Among these categories, alkaloids and derivatives accounted for a relatively high proportion in all samples, but their relative abundance was generally higher in younger samples and decreased in samples stored for 9 to 25 years. In contrast, the relative proportion of phenylpropanoids and polyketides increased significantly in samples aged for 9 to 25 years, indicating that long-term storage is accompanied by continuous remodeling of phenylpropanoid- and polyketide-related metabolic networks. Meanwhile, lipids and lipid-like molecules, organic acids and derivatives, and organoheterocyclic compounds also showed varying degrees of fluctuation across storage years. Combined with the stacked plot of absolute abundance (Figure S1), it can be further observed that different metabolite categories not only underwent changes in compositional proportions, but also exhibited significant differences in overall signal intensity during storage. These results indicate that the long-term aging of QLT is not characterized by the unidirectional increase or decrease of a single metabolite, but rather by the synergistic reconstruction of multiple non-volatile components [7].

Unsupervised analysis further revealed the overall separation pattern among samples. The PCA score plot showed that PC1 and PC2 explained 47.82% and 11.74% of the total variance, respectively (Figure 2C). Samples from different storage years showed clear separation in the score plot. Overall, Q2 and Q4 were located in the left region of the plot, whereas Q6, Q9, and Q12 were mainly distributed in the intermediate transitional zone, while Q14, Q18, Q20, and Q25 were more frequently distributed in the right region. This distribution pattern indicates that QLT undergoes pronounced stage-dependent metabolic remodeling during long-term storage. The results of hierarchical clustering analysis were generally consistent with the separation pattern revealed by PCA (Figure 2E). Q2 and Q4 showed high similarity, with Q6, Q9, and Q12 clustered as an intermediate group, whereas Q14, Q18, Q20, and Q25 were grouped together as a later-stage cluster. To further improve the visualization of sample separation, PLS-DA was also performed. In the supervised model, samples from different storage years showed a certain degree of separation, indicating that storage duration can, to some extent, drive differences in metabolic profiles (Figure 2D). The permutation test results showed that, although the model outperformed the randomly permuted models, no significant overfitting was observed (Figure S2).

Overall, the untargeted metabolomic results indicate that the non-volatile metabolic profile of QLT undergoes significant remodeling during long-term storage. Samples stored for different years exhibited marked differences in metabolite category composition, overall profile distribution, and clustering relationships. The samples were further grouped into three storage stages: Q2 and Q4 as the early stage, Q6, Q9, and Q12 as the middle stage, and the remaining samples as the late stage. The early stage is characterized by rapid stabilization, involving the decrease in residual moisture and the oxidative conversion of catechins. During the middle stage, continuous oxidation and polymerization convert astringent polyphenols into stable pigments. In the late stage, the chemical composition reaches a relatively stable equilibrium, accompanied by the gradual formation of aged-related aroma compounds. This classification is broadly consistent with previous findings [12,16]. Notably, the assignment of early, middle, and late stages is defined relative to the twenty-five-year storage window examined in this study, reflecting the stage-dependent dynamic maturation process of QLT. These results provide an important basis for subsequent differential metabolite screening and metabolic pathway enrichment analysis.

### 3.3. Differential Metabolites Profiling Among Storage-Stage Groups

**Figure 3:**
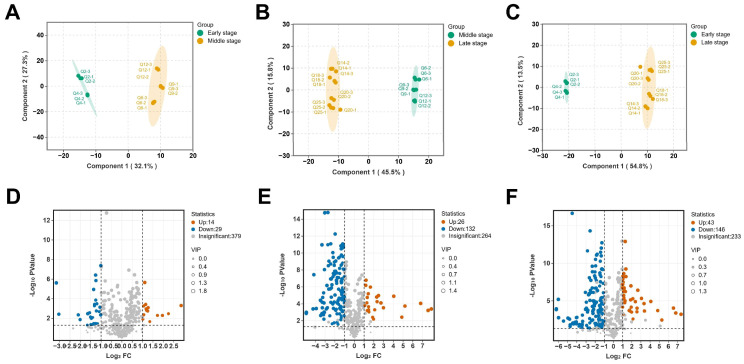
OPLS-DA and volcano plot analysis of differential non-volatile metabolites among different storage stages of QLT. (**A**) OPLS-DA score plot of the early stage vs. middle stage comparison. (**B**) OPLS-DA score plot of the middle stage vs. late stage comparison. (**C**) OPLS-DA score plot of the early stage vs. late stage comparison. (**D**) Volcano plot of the early stage vs. middle stage comparison. (**E**) Volcano plot of the middle stage vs. late stage comparison. (**F**) Volcano plot of the early stage vs. late stage comparison.

To further elucidate the chemical differences in QLT during long-term storage, pairwise OPLS-DA and univariate differential analyses were performed for the early vs. middle, middle vs. late, and early vs. late stages. As shown in the score plot (Figure 3A–C), the samples in each comparison were clearly separated, indicating significant stage-dependent differences in non-volatile metabolites. Notably, the separation between the middle and late stages, as well as that between the early and late stages, was more pronounced than that between the early and middle stages. According to the screening criteria of VIP > 1, *p* < 0.05, and |log_2_FC| > 1, 43, 158, and 189 differential metabolites were identified in the comparisons of the early vs. middle, middle vs. late, and early vs. late stages, respectively (Figure 3D–F). Among them, 14 metabolites were upregulated and 29 were downregulated in the comparison between the early and middle stages. In the comparison between the middle and late stages, 26 metabolites were upregulated and 132 were downregulated. In the comparison between the early and late stages, 43 metabolites were upregulated and 146 were downregulated. These results indicate that the number of differential metabolites increased markedly with prolonged storage, while the number of downregulated metabolites consistently exceeded that of upregulated metabolites, particularly in comparisons involving the later stages. This finding is consistent with previous studies on aged dark tea, in which long-term storage was mainly manifested as the depletion, transformation, or accumulation of non-volatile compounds [4,6].

**Figure 4:**
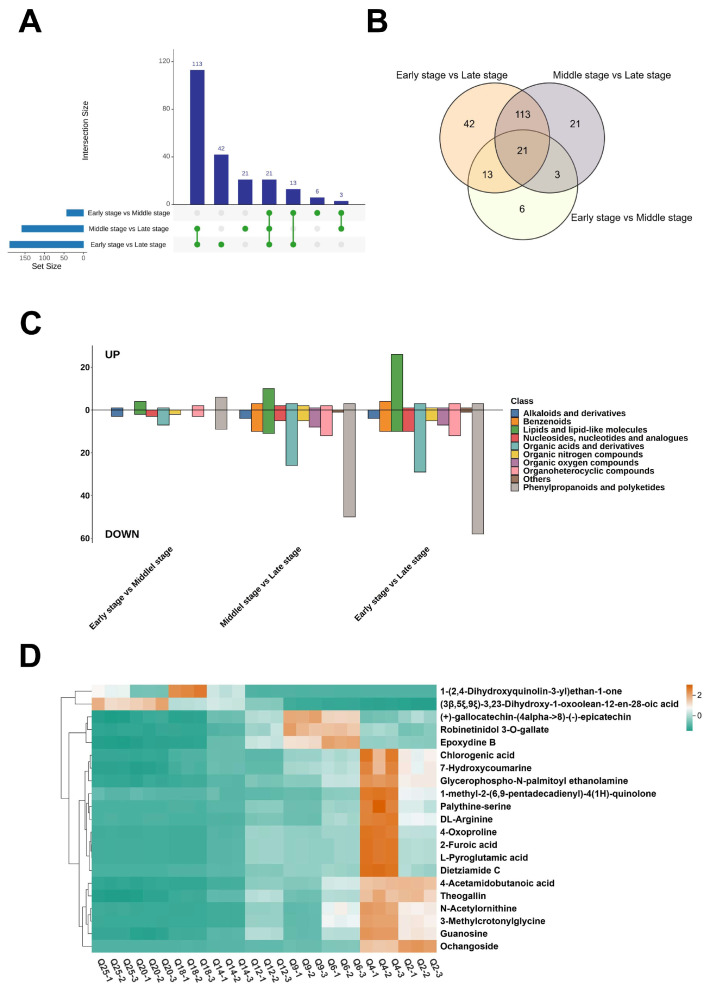
Comparative analysis of differential metabolites among different storage-stages of QLT. (**A**) UpSet plot showing the overlap of differential metabolites among pairwise comparisons of storage-stages. (**B**) Venn diagram of shared and unique differential metabolites among different storage stage comparisons. (**C**) Classification of differential metabolites based on chemical categories across different storage stage comparisons. (**D**) Hierarchical clustering heatmap of 21 shared differential metabolites among the three storage stages.

To further summarize the overlap and stage specificity of differential metabolites, UpSet and Venn analyses were performed (Figure 4A,B). After merging and removing duplicates from all pairwise differential metabolites, a total of 219 unique differential metabolites were obtained. Among them, 21 differential metabolites were shared across all three pairwise comparisons, indicating that these compounds may represent the core metabolic features associated with storage-stage transitions. In addition, the overlap between the middle-to-late and early-to-late stage comparisons reached 113 metabolites, which was markedly higher than that of the other pairwise comparisons. Meanwhile, 42, 21, and 6 stage-specific metabolites were identified in the early-to-late, middle-to-late, and early-to-middle comparisons, respectively. These findings further support the view that the late stage possesses the most distinct chemical characteristics and that the transition from the middle to late stage represents a critical period for metabolite remodeling. Similar phenomena have also been observed in long-term stored Liupao tea and White tea, where the accelerated remodeling of non-volatile metabolites related to polyphenols and taste during the later storage period was associated with changes in quality formation [4,17].

As shown in Figure 4C, the differential metabolites mainly belonged to alkaloids and derivatives, benzenoids, lipids and lipid-like molecules, nucleosides, nucleotides and analogues, organic acids and derivatives, organic nitrogen compounds, organic oxygen compounds, organoheterocyclic compounds, and phenylpropanoids and polyketides. Among these categories, phenylpropanoids and polyketides, organic acids and derivatives, and lipids and lipid-like molecules were the major classes of differential metabolites. Previous studies have shown that these three classes of compounds are widely considered important contributors to taste attributes such as astringency, freshness, mellowness, and aged sensory characteristics [18,19,20].

Within the lipid and lipid-like molecule category (Figure S3A), 42 differential metabolites were identified. Specifically, 24 compounds showed a gradual increase from the early to late stages, whereas 10 compounds exhibited a sustained decrease, and 6 compounds accumulated preferentially during the middle stage. Representative compounds such as 1,4-androstadiene-3,17-dione, 22α-hydroxyerythrodiol, and (3*β*,5*ξ*,9*ξ*)-3,23-dihydroxy-1-oxoolean-12-en-28-oic acid (DOA) increased markedly with storage, suggesting the gradual accumulation of certain triterpenoid- or steroid-like lipid components during aging. In contrast, dioxolide B, 2-methoxyestradiol, 12-oxo-phytodienoic acid, linoleoyl ethanolamide, and glycerophospho-N-palmitoyl ethanolamine showed marked declines from the early to late stages. Given that lipid oxidation and membrane-associated lipid turnover can influence flavor evolution and storage-related matrix changes, these findings suggest that lipid remodeling represents an important biochemical event during QLT aging [18,21].

Within the category of organic acids and derivatives, a total of 35 compounds were identified (Figure S3B), most of which showed a decreasing trend during storage. Among them, 26 compounds decreased continuously from the early to late stages, whereas only 2 compounds showed a sustained increase. In addition, 5 compounds exhibited relatively high abundance during the middle stage of storage. Representative downregulated compounds included N-acetylornithine, 3-methylcrotonylglycine, 4-acetamidobutanoic acid, 4-oxoproline, L-pyroglutamic acid, L-aspartic acid, and L-phenylalanine. Many of these compounds are closely associated with amino acid degradation, nitrogen metabolism, and the coordination of tea flavor [22]. Only a few compounds, such as 5-hydroxytryptophan and hexadecanedioic acid, showed a significant increase during the later stages of storage. Previous studies have suggested that decreases in amino acid-related acids and other polar acidic metabolites are associated with reduced astringency and freshness, together with enhanced mellowness or aged flavor characteristics [6,22].

Phenylpropanoids and polyketides represented the largest of the three key metabolite classes (Figure S3C), comprising 74 compounds, and showed the most pronounced response to storage. Among these compounds, 56 declined continuously from the early to late storage stages, 13 exhibited a middle-stage accumulation pattern, and only 5 gradually increased throughout storage. Representative downregulated compounds included ochangoside, theasinensin C, chlorogenic acid, 7-hydroxycoumarin, and catechin-related metabolites. In contrast, a limited number of compounds, such as glycitein, genistein, scoparone, and 7,8-dihydroxy-4-methylcoumarin, increased during storage. Notably, (+)-gallocatechin-(4*α*→8)-(-)-epicatechin and robinetinidol 3-O-gallate were relatively abundant at the middle stage of storage, suggesting that the oligomerization and transformation of flavan-3-ols may be particularly active during the transition toward more advanced aging. Given that phenylpropanoid- and flavonoid-derived metabolites are major determinants of tea bitterness, astringency, mouthfeel, and aged character, their extensive depletion and structural reorganization likely constitute the principal chemical basis underlying the evolution of infusion quality during QLT storage. This interpretation is further supported by recent studies on Longjing tea, Liupao tea, Pu-erh tea, and aged white tea, in which phenolic acids, catechins, flavonoid glycosides, and polymeric phenolics have repeatedly been identified as key non-volatile drivers of sensory changes [4,6,20,22].

Based on the UpSet and Venn analyses, heatmap analysis of the 21 shared differential metabolites further substantiated the central role of the three key metabolite classes in discriminating storage stages (Figure 4D). Among these 21 shared metabolites, 13 belonged to the three major classes described above, including two lipids and lipid-like molecules, five organic acids and derivatives, and six phenylpropanoids and polyketides. Most of these shared differential metabolites, such as glycerophospho-N-palmitoyl ethanolamine, N-acetylornithine, 3-methylcrotonylglycine, 4-acetamidobutanoic acid, 4-oxoproline, L-pyroglutamic acid, chlorogenic acid, 7-hydroxycoumarin, theogallin, and ochangoside, showed a general declining trend during storage. In contrast, DOA increased progressively, whereas (+)-gallocatechin-(4*α*→8)-(-)-epicatechin and robinetinidol 3-O-gallate accumulated during the middle stage before declining thereafter. Together, these patterns suggest that QLT storage involves a stagewise reorganization process characterized by the concurrent depletion, accumulation, and transformation of non-volatile compounds.

Collectively, the results of pairwise comparisons, overlap analysis, class distribution, and class-specific heatmap analyses consistently demonstrate that long-term storage induces extensive remodeling of the non-volatile metabolome in QLT, with the most pronounced differences emerging at the late storage stage. In particular, phenylpropanoids and polyketides, organic acids and derivatives, and lipids and lipid-like molecules represent the primary metabolic basis for discriminating storage stages and are likely the major chemical contributors to changes in infusion quality during storage. These findings provide a solid foundation for subsequent pathway enrichment analysis and for elucidating the relationship between metabolomic remodeling and sensory quality attributes.

### 3.4. Pathway Remodeling Associated with Storage

**Figure 5:**
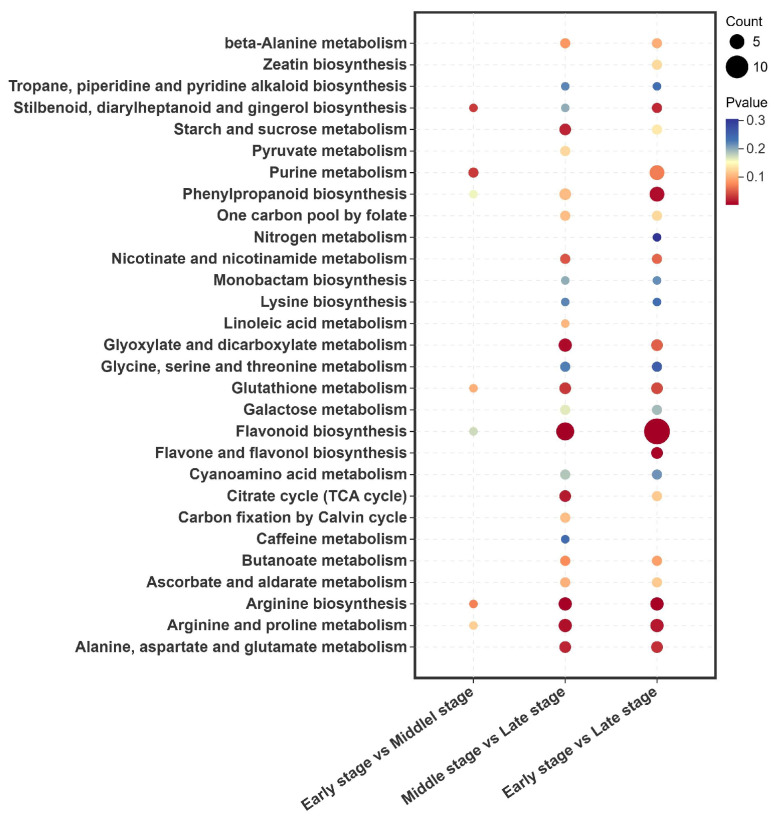
Combined KEGG enrichment bubble plot of differential metabolites among the three storage-stage comparisons of QLT.

To further elucidate the biochemical significance underlying storage-stage discrimination, KEGG enrichment analysis was performed based on the differential metabolites identified in the three pairwise comparisons. The enrichment bubble plot (Figure 5) showed that the metabolic remodeling associated with QLT storage involved coordinated shifts in flavonoid and phenylpropanoid metabolism, amino acid and nitrogen metabolism, carbohydrate and energy metabolism, as well as several alkaloid- and lipid-related pathways. A pronounced stage effect was evident in the breadth of pathway enrichment. Only seven pathways were enriched in the comparison between the early and middle stages, including stilbenoid, diarylheptanoid and gingerol biosynthesis, purine metabolism, arginine biosynthesis, glutathione metabolism, arginine and proline metabolism, phenylpropanoid biosynthesis, and flavonoid biosynthesis. In contrast, 46 and 44 pathways were enriched in the middle-to-late and early-to-late comparisons, respectively. These findings suggest that metabolic reprogramming intensified substantially with prolonged storage, particularly after the middle stage.

Among all enriched pathways, flavonoid biosynthesis emerged as the strongest and most consistent signal. Its enrichment intensity increased markedly from the early-to-middle transition to the comparisons involving the late stage, with 1, 7, and 12 metabolites assigned to this pathway in the early vs. middle, middle vs. late, and early vs. late comparisons, respectively. More importantly, after multiple-testing correction, flavonoid biosynthesis remained significantly enriched in the middle vs. late comparison (adjusted *p* = 0.0162) and the early vs. late comparison (adjusted *p* = 2.26 × 10^−7^), identifying it as the most statistically robust pathway associated with storage progression in this study. Previous studies have shown that remodeling of the flavonoid pathway is a key driver of changes in bitterness, astringency, mouthfeel, and overall infusion quality [18,20,21].

Parallel to flavonoid biosynthesis, phenylpropanoid biosynthesis was enriched in all three comparisons, with the number of mapped metabolites increasing from 1 in the early vs. middle comparison to 3 in the middle vs. late comparison and 5 in the early vs. late comparison. The consistent enrichment of this pathway across all storage transitions suggests that phenolic precursor remodeling is stably involved in tea aging. Given that the phenylpropanoid pathway supplies precursors for flavonoids, phenolic acids, and related polymeric products, its sustained enrichment further implies that storage-induced changes in infusion quality are closely associated with the redistribution and transformation of the phenolic framework [1,17,18].

Another notable feature was the persistent enrichment of the arginine biosynthesis and arginine and proline metabolism pathways, both of which were detected in all three comparisons. The number of associated metabolites increased from 1 in the early vs. middle comparison to 4 in each of the latter two comparisons. In addition, several other amino acid-related pathways, including alanine, aspartate and glutamate metabolism, glycine, serine and threonine metabolism, lysine biosynthesis, and cyanoamino acid metabolism, were mainly enriched in the comparisons involving the late stage. These findings suggest that storage progressively reshapes nitrogen redistribution and amino acid-derived metabolism in QLT. Given that amino acids and their derivatives contribute to umami, sweetness, smoothness, and overall taste harmony, changes in these pathways are likely involved in the sensory transition of the tea infusion from relative freshness and briskness toward a mellower and more aged character during prolonged storage [6,22].

Compared with the early vs. middle-stage comparison, the late-stage comparisons additionally involved a broader range of carbon and energy metabolism pathways, including starch and sucrose metabolism, galactose metabolism, pyruvate metabolism, glyoxylate and dicarboxylate metabolism, the citrate cycle, and one-carbon pool by folate. The enrichment of these pathways suggests that storage-associated chemical evolution is accompanied by the reorganization of central carbon flux and organic acid metabolism. Such changes may influence both the direct accumulation of taste-active organic acids and the indirect formation of secondary metabolites derived from primary metabolic precursors [17,21]. Notably, seven pathways were shared across all three pairwise comparisons, namely flavonoid biosynthesis, phenylpropanoid biosynthesis, stilbenoid, diarylheptanoid and gingerol biosynthesis, purine metabolism, arginine biosynthesis, arginine and proline metabolism, and glutathione metabolism. These shared pathways likely represent the core biochemical framework underlying storage-stage transitions in QLT. However, the substantially greater number of pathways detected in the middle vs. late and early vs. late comparisons, relative to the early vs. middle comparison, suggests that the late storage stage is characterized not simply by a continuation of earlier trends, but by broader metabolic reprogramming involving both primary and secondary metabolism. In other words, the transition from moderately aged to aged QLT appears to represent a critical window for pathway remodeling and quality formation.

Overall, KEGG enrichment analysis indicated that QLT storage was accompanied by progressive and stage-dependent pathway remodeling. The earliest storage transition involved a relatively limited set of enriched pathways, whereas late-stage storage triggered broader metabolic reprogramming centered on flavonoid biosynthesis, together with coordinated shifts in phenylpropanoid metabolism, amino acid and nitrogen metabolism, redox-related metabolism, and central carbon metabolism. These pathway-level alterations provided a crucial mechanistic basis for the extensive non-volatile metabolomic reorganization described in Section 3.3 and likely underpinned the evolution of infusion quality during storage. Collectively, the pathways highlighted herein provide a biochemical bridge linking storage duration to changes in key metabolite classes and, ultimately, to the sensory and quality transformations characteristic of aged QLT.

### 3.5. Sensory Evolution of QLT Across Storage Years

**Figure 6:**
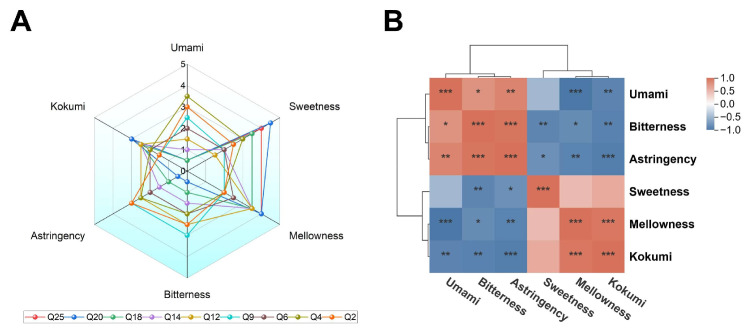
QDA radar plot and correlation analysis of sensory attributes in QLT with different storage years. (**A**) Radar plot of umami, sweetness, mellowness, bitterness, astringency, and kokumi. (**B**) Correlation heatmap of the six sensory attributes. In subfigure (**B**), asterisks indicate statistical significance: * *p* < 0.05, ** *p* < 0.01, and *** *p* < 0.001.

To further characterize the evolution of infusion quality in QLT during storage, sensory evaluation was performed using QDA, with six sensory attributes selected: umami, sweetness, mellowness, bitterness, astringency, and kokumi. The radar plot (Figure 6A) revealed a distinct sensory trajectory across storage years, showing that the taste profile of QLT gradually shifted from a relatively fresh, brisk, and bitter-astringent style in the early storage stage toward a sweeter, mellower, and fuller-bodied character in the late stage. Notably, the middle stage appeared to represent a critical sensory turning point during QLT aging. Although middle-stage samples still retained some freshness and bitter-astringent characteristics typical of young tea, their mellowness and kokumi attributes had already begun to increase, suggesting that the infusion was evolving toward a rounder and more mature taste profile. This transitional pattern was highly consistent with the metabolomic findings presented in Section 3.3 and Section 3.4, in which the extent of differential metabolite changes and pathway remodeling revealed in the middle vs. late comparison far exceeded that observed in the early vs. middle comparison. Collectively, these results suggest that sensory evolution and metabolic remodeling were tightly coupled, and that the transition from the middle to late stage constituted a critical window for infusion quality formation during storage [16,21].

The correlation clustering heatmap of QDA attributes further revealed the internal sensory structure of QLT (Figure 6B). Bitterness and astringency exhibited a very strong positive correlation with each other, and both were positively correlated with umami, whereas these three attributes were negatively correlated with sweetness, mellowness, and kokumi. In contrast, sweetness, mellowness, and kokumi formed another highly intercorrelated cluster, suggesting that these desirable sensory attributes improved synchronously during storage. From the perspective of infusion quality, these findings indicate that storage reshaped QLT primarily by weakening coarse, pungent, and brisk sensations while enhancing perceptions related to sweetness, smoothness, and body. This sensory shift is of considerable significance for aged dark tea, as preferences for aged tea among both consumers and trained panelists are often associated with reduced astringency and irritation, together with increased smoothness, mellowness, and harmonious mouthfeel [16,23].

Overall, sensory evaluation demonstrated that QLT underwent pronounced taste evolution during storage. Young samples were characterized by prominent umami, bitterness, and astringency, whereas aged samples were distinguished by elevated sweetness, mellowness, and kokumi. The middle stage represented a clear transitional state, and the attribute-correlation heatmap further revealed that the six QDA attributes were organized into two opposing sensory modules. Collectively, these findings provide an important sensory basis for subsequent correlation analysis between key non-volatile metabolites and taste attributes.

### 3.6. Associations Between Key Non-Volatile Metabolites and Taste Attributes

**Figure 7:**
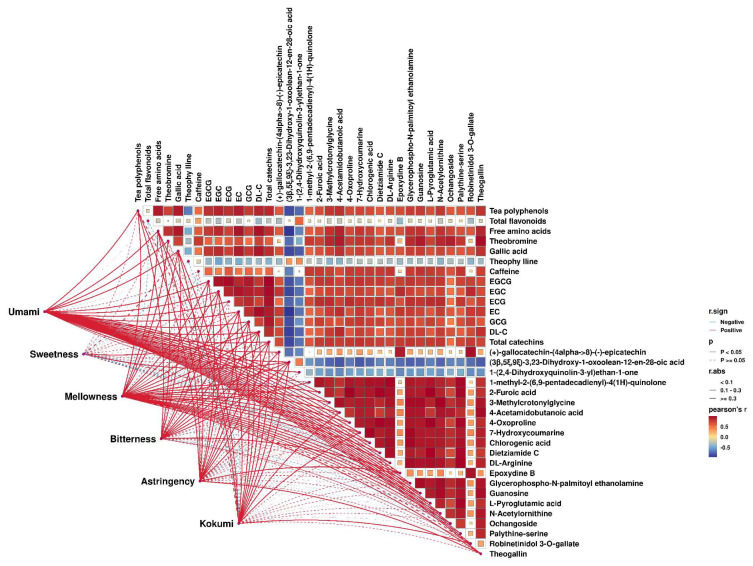
Correlation heatmap between QDA sensory attributes and key non-volatile compounds in QLT. Red and blue lines indicate positive and negative correlations, respectively, while solid and dashed lines represent significant and non-significant correlations, respectively.

Multivariate sensory-chemical coupling analysis has emerged as a key strategy for identifying taste-active drivers in tea quality research [10]. To elucidate the chemical basis underlying the sensory evolution of QLT during storage, Pearson correlation analysis was performed between the six QDA taste attributes and key non-volatile variables, including physicochemical indices, catechins, and representative shared differential metabolites selected from Section 3.3. The integrated correlation heatmap and network diagram (Figure 7) revealed that the associations between taste attributes and chemical variables were highly structured rather than randomly distributed, with most significant correlations being positive, whereas negative correlations were generally weak and largely non-significant.

Among the six sensory attributes, umami exhibited the highest number of strong associations, suggesting that it was the most chemically responsive taste dimension in QLT. This attribute was primarily driven by catechin monomers and phenolic acids, with DL-C (r = 0.908, *p* = 0.001), EC (r = 0.902, *p* = 0.001), and gallic acid (r = 0.891, *p* = 0.002) displaying the most prominent positive correlations. Additional significant contributors included free amino acids, EGC, total catechins, tea polyphenols, and DOA (r = 0.758, *p* = 0.001). Together, these findings suggest that umami in stored QLT was jointly regulated by amino acid-related compounds, catechin-related variables, and storage-responsive differential metabolites. Similar multi-component regulation of umami has also been reported in previous studies, in which amino acids, catechins, and phenolic acids collectively contribute to perceived taste balance [16,24]. Conversely, sweetness exhibited the weakest direct chemical associations among the six QDA attributes. Only the DOA (r = 0.382, *p* = 0.030) and DL-C (r = 0.380, *p* = 0.038) showed significant positive correlations with this trait. Such a limited number of direct drivers suggests that sweetness perception in stored QLT may arise less from individual compounds than from the overall sensory balance created by the attenuation of bitterness and astringency alongside the enhancement of mellowness and kokumi [16,24].

Mellowness exhibited extensive and significant positive correlations with both conventional quality indices and differential metabolites. The development of this sensory trait was highly dependent on EC (r = 0.776, *p* = 0.005) and the DOA (r = 0.710, *p* = 0.006). Furthermore, EGC, 7-hydroxycoumarin, free amino acids, and DL-C all provided substantial contributions to this attribute. In addition, chlorogenic acid, gallic acid, GCG, and several organic acid-related metabolites also showed significant positive correlations with mellowness. Collectively, these results suggest that the development of a mellow mouthfeel in QLT depends on coordinated changes among catechins, phenolic acids, amino acid-related compounds, and storage-derived differential metabolites [24,25,26].

Consistent with their strong mutual relationship in the QDA heatmap, bitterness and astringency displayed nearly parallel chemical correlation profiles. Both attributes were profoundly influenced by the DOA, which yielded the highest correlation coefficients for both bitterness (r = 0.822, *p* = 0.002) and astringency (r = 0.850, *p* = 0.004). Other prominent contributors to these two sensory dimensions included gallic acid, DL-C, EC, and free amino acids, all of which exhibited correlation coefficients exceeding 0.670. Significant positive correlations were additionally observed for tea polyphenols, total catechins, EGC, GCG, and EGCG. These results align well with previous studies confirming that catechins, tea polyphenols, and gallic acid derivatives serve as key chemical drivers of bitterness and astringency in tea infusions [20,24,27].

Regarding kokumi, the correlation results suggest that this pleasant, body-related sensory dimension is governed by a mixed set of conventional compounds and storage-responsive metabolites. Interestingly, this attribute was characterized by prominent negative correlations with several variables, particularly the DOA (r = -0.652, *p* = 0.002), EC (r = -0.645, *p* = 0.003), DL-C, free amino acids, gallic acid, and EGC. Conversely, significant positive associations were identified between kokumi and specific differential metabolites, including 4-acetamidobutanoic acid, (+)-gallocatechin-(4*α*→8)-(-)-epicatechin, 7-hydroxycoumarin, and robinetinidol 3-O-gallate.

In summary, the sensory–chemical correlation analysis revealed that the taste attributes of QLT were closely associated with coordinated changes in key non-volatile compounds. Umami, bitterness, and astringency were strongly associated with catechin- and polyphenol-related variables, as well as specific shared differential metabolites. In contrast, mellowness and kokumi were influenced by a wider range of amino acid-related compounds, phenolic compounds, and storage-responsive metabolites. Sweetness, however, exhibited comparatively limited direct correlations. Overall, these findings provide robust empirical evidence for the chemical basis underlying the sensory evolution of QLT during long-term storage. However, as all samples were obtained from a single manufacturer and stored under non-controlled natural warehouse conditions, the observed metabolic changes cannot be attributed solely to storage duration, and the generalizability of these findings is inherently limited. Therefore, the compounds identified in this study should be regarded as candidate markers associated with storage. Further confirmation is required in subsequent studies that incorporate microbial metabolism research, as well as broader multi-site and longitudinal investigations.

## 4. Conclusions

Anhua Qianliang tea (QLT), a representative compressed dark tea, undergoes continuous quality transformation during long-term storage, yet the material basis of its non-volatile quality evolution remains unclear. This study systematically elucidated the storage-year-dependent evolution of non-volatile quality in QLT by integrating physicochemical analysis, targeted catechin quantification, untargeted metabolomics and QDA sensory evaluation. Long-term storage induced pronounced remodeling of both the basic chemical composition and the global non-volatile metabolome of QLT. Total polyphenols, free amino acids, and major catechin fractions generally declined with prolonged storage. Differential metabolite and KEGG pathway analyses demonstrated that phenylpropanoids and polyketides, organic acids and derivatives, and lipids and lipid-like molecules were the major metabolite classes associated with storage-stage discrimination, while flavonoid biosynthesis, phenylpropanoid biosynthesis, amino acid-related metabolism, and central carbon metabolism represented the core pathways underlying storage-associated metabolic remodeling. Sensory analysis further revealed that QLT underwent a distinct taste transition during storage, shifting from a younger profile characterized by higher umami, bitterness, and astringency toward an aged profile with enhanced sweetness, mellowness, and kokumi. Correlation analysis confirmed that this sensory evolution was closely linked to coordinated changes in catechins, polyphenols, phenolic acids, amino acid-related metabolites, and several representative storage-responsive differential metabolites. Overall, these findings provide new insights into the chemical basis of non-volatile quality evolution in QLT during long-term storage and offer a theoretical basis for quality evaluation and storage-age assessment.

## Data Availability

The original contributions presented in this study are included in the article/Supplementary Materials. Further inquiries can be directed to the corresponding author.

## Supplementary Materials

The following supporting information can be downloaded at: https://www.mdpi.com/article/10.3390/foods15132376/s1, Figure S1: Stacked bar plot of absolute abundance showing the overall signal intensity of different non-volatile metabolite categories in QLT across various storage years; Figure S2: Permutation test results validating the partial least squares discriminant analysis model. The plot demonstrates the R2Y and Q2 statistical parameters which indicate the absence of significant overfitting for the classification of QLT samples across different storage years; Figure S3: Hierarchical clustering heatmaps showing the relative abundance of the three major classes of differential metabolites in QLT across different storage years. (A) Lipids and lipid-like molecules. (B) Organic acids and derivatives. (C) Phenylpropanoids and polyketides. The color scale represents the Z score transformed relative abundance of each metabolite. Table S1: Conventional physicochemical indices and major catechin compositions of QLT across different storage years; Table S2: Identified non-volatile metabolites in QLT; Table S3: Key discriminative non-volatile metabolites of QLT during aging; Table S4: KEGG analysis of differential metabolites; Table S5: Pearson correlation parameters between quantitative descriptive analysis sensory attributes and key non-volatile chemical compounds in QLT across different storage years; Table S6: Identification of key non-volatile metabolites highly correlated with the storage duration pattern of QLT via Pattern Hunter analysis. Data include the correlation coefficient t statistic nominal *p*-value and false discovery rate for each specific compound.

## Abbreviations

The following abbreviations are used in this manuscript:

QLT	Anhua Qianliang tea
QDA	Quantitative descriptive analysis
ROC	Receiver operating characteristic
GA	Gallic acid
EGCG	(-)-Epi-gallocatechin gallate
ECG	(-)-Epicatechin gallate
EGC	(-)-Epi-gallocatechin
EC	(-)-Epicatechin
DL-C	(+)-Catechin
GCG	(+)-gallocatechin-3-O-gallate
FAAs	Free amino acids
TPs	Tea polyphenols
HPLC	High-performance liquid chromatography
OPLS-DA	Orthogonal partial least squares-discriminant analysis
PLS-DA	Partial least squares-discriminant analysis
PCA	Principal component analysis
DOA	(3*β*,5*ξ,9ξ*)-3,23-dihydroxy-1-oxoolean-12-en-28-oic acid
