# Supplementary material for: Non-Volatile Metabolic Reprogramming and Sensory Evolution of Anhua Qianliang Tea During Long-Term Storage
Source: Foods. 2026 Jul 3;15(13):2376. doi: 10.3390/foods15132376 (PMC13361243; doi:10.3390/foods15132376)
Supplement: Supplementary file 1 [file foods-15-02376-s001.zip › Supplementary figures.pdf]

Supplemental Figure Captions:

Figure S1. Stacked bar plot of absolute abundance showing the overall signal intensity of different non volatile metabolite categories in QLT across various storage years.

Figure S2. Permutation test results validating the partial least squares discriminant analysis model. The plot demonstrates the R2Y and Q2 statistical parameters which indicate the absence of significant overfitting for the classification of QLT samples across different storage years.

Figure S3. Hierarchical clustering heatmaps showing the relative abundance of the three major classes of differential metabolites in QLT across different storage years. (A) Lipids and lipid like molecules. (B) Organic acids and derivatives. (C) Phenylpropanoids and polyketides. The color scale represents the Z score transformed relative abundance of each metabolite.

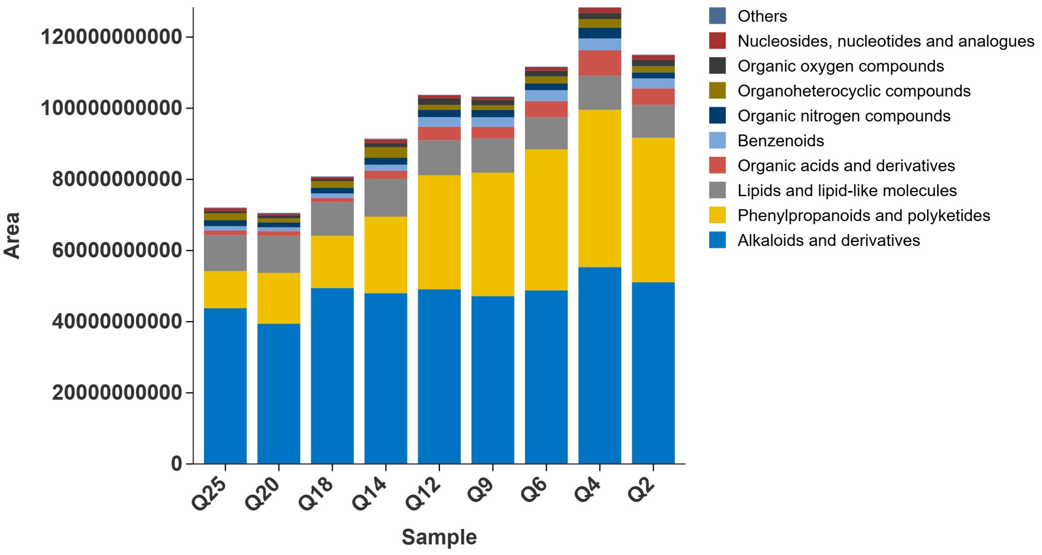

Figure S1. Stacked bar plot of absolute abundance showing the overall signal intensity of different non volatile metabolite categories in QLT across various storage years.

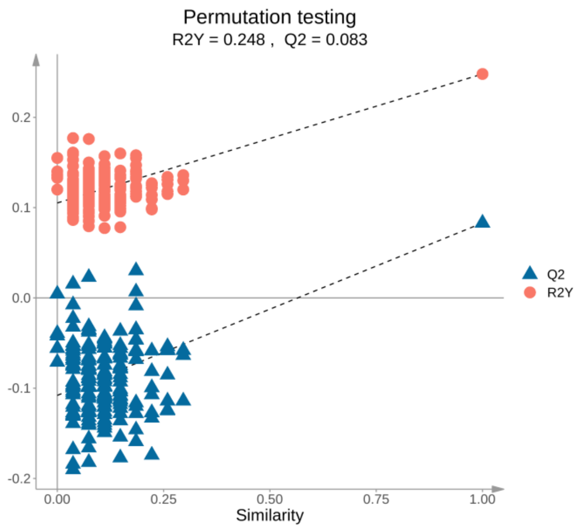

Figure S2. Permutation test results validating the partial least squares discriminant analysis model. The plot demonstrates the R2Y and Q2 statistical parameters which indicate the absence of significant overfitting for the classification of QLT samples across different storage years.

A

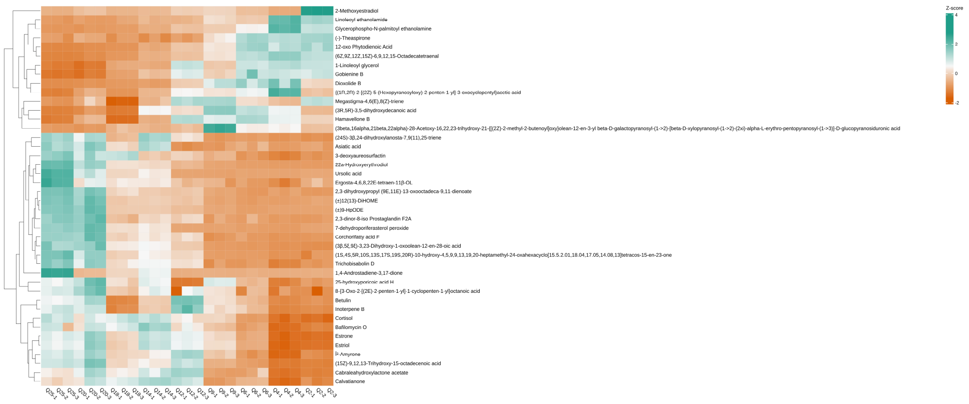

B

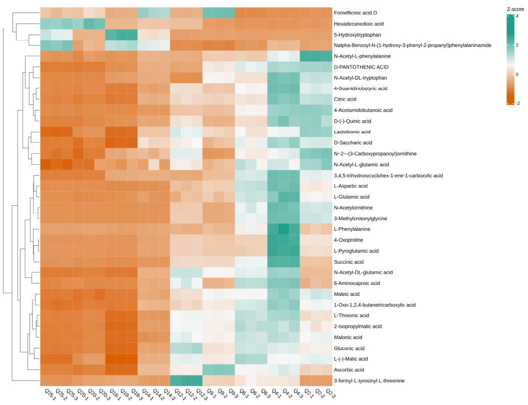

C

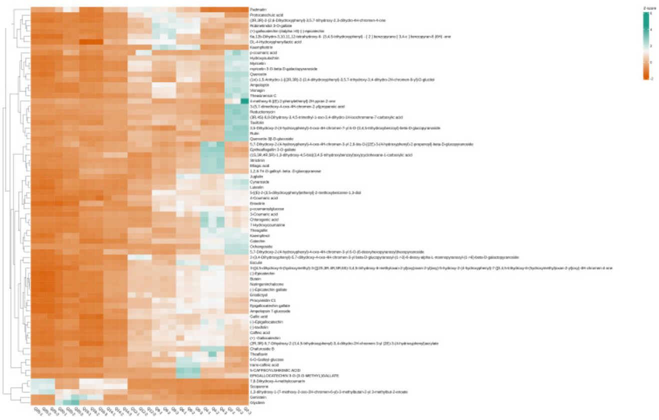

Figure S3. Hierarchical clustering heatmaps showing the relative abundance of the three major classes of differential metabolites in QLT across different storage years. (A) Lipids and lipid like molecules. (B) Organic acids and derivatives. (C) Phenylpropanoids and polyketides. The color scale represents the Z score transformed relative abundance of each metabolite.
